# Effects of Hypoxia on Biology of Human Leukemia T-cell Line (MOLT-4 cells) Co-cultured with Bone Marrow Mesenchymal Stem Cells

**Published:** 2018

**Authors:** Sina Baharaghdam, Mehdi Yousefi, Aliakbar Movasaghpour, Saeed Solali, Mehdi Talebi, Milad Ahani-Nahayati, Hamid Lotfimehr, Karim Shamsasanjan

**Affiliations:** 1. Immunology Research Center, Tabriz University of Medical Sciences, Tabriz, Iran; 2. Hematology and Oncology Research Center, Tabriz University of Medical Sciences, Tabriz, Iran; 3. Stem Cell and Regenerative Medicine Institute, Tabriz University of Medical Sciences, Tabriz, Iran; 4. Department of Immunology, Faculty of Medicine, Tabriz University of Medical Sciences, Tabriz, Iran

**Keywords:** Acute lymphoblastic leukemia, Drug resistance, Hypoxia, Mesenchymal stem cell

## Abstract

**Background:**

One of the most significant problems in the treatment of leukemia is the expansion of resistance to chemotherapeutic agents. Therefore, assessing the drug resistance and especially the drug resistance genes of leukemic cells is important in any treatment. The impact of Mesenchymal Stem Cells (MSCs) and hypoxic condition have been observed in the biological performance of majority of leukemic cells.

**Methods:**

MOLT-4 cells were co-cultured with MSCs in the hypoxic condition induced by Cobalt Chloride (CoCl_2_) for 6 and 24 *hr*. Then, apoptosis of cells was analyzed using annexin-V/PI staining and expression of the drug resistance genes including MDR1, MRP, and BCRP along with apoptotic and anti-apoptotic genes, including BAX and BCL2, was evaluated by real-time PCR.

**Results:**

The hypoxic condition for MOLT-4 cells co-cultured with MSCs could significantly increase the expression of MDR1 and BCRP genes (p<0.05) which are involved in drug resistance. Also, the results indicated that this condition significantly increases the expression of BCL2 (p<0.05) and reduces the apoptosis in MOLT-4 cells co-cultured with MSCs in the hypoxic condition.

**Conclusion:**

These effects can demonstrate the important role of hypoxia and MSCs on the biological behavior of Acute Lymphoblastic Leukemia (ALL) cells that may lead to particular treatment outcomes.

## Introduction

Childhood malignancies are serious problems that can cause inexpiable damages to families and societies. About 35% of childhood malignancies are childhood leukemia and Acute Lymphoblastic Leukemia (ALL) involves 80–85% of them^1^. Mesenchymal Stem Cells (MSCs) are multipotent stromal cells that can differentiate into a variety of cells and have a critical role in hematopoiesis regulation and successful hematopoietic stem cell transplantation. There is some evidence that the MSCs have a supporting effect on tumor stroma through various activities such as producing growth factors, amplifying tumor vessel formation and forming niches for tumor stem cells especially in solid tumors like gastric and breast cancer. Thus, it shows that MSCs of tumor microenvironment can promote tumor growth in several ways^2^. Besides, it has always been debatable that how the MSCs influence the leukemic cells. In some studies, apoptosis induction was supposed to be an answer to this question^3^. Several studies have shown a proliferation-suppressing effect of MSCs on lymphoid lineage by secreting multiple cytokines that arrest these cells at G0 and G1 phases of cell cycle^4^. Therefore, more investigations are needed to clarify the MSCs effects in lymphoid leukemia.

Hypoxia-Inducible Factor-1 (HIF-1), a heterodimeric transcription factor, is an essential part of the cellular response to the hypoxic condition^5^. This element was swiftly destroyed under normoxic condition via proteasomal degradation, but in the hypoxic situation, its degradation is slow, which eventually leads to increased expression of its target genes. Cobalt chloride (CoCl_2_) has the potential of blocking the HIF-1 degradation and hence it can mimic the hypoxic situation^6^. Based on many reliable reports in several different kinds of cells, CoCl_2_ can induce apoptosis^7–10^.

It is a big challenge to prevent leukemic cells recurrence in the bone marrow to overcome treatment failure in ALL. Bone marrow environment is heterogeneous and has a hypoxic compartment^11–13^. As a result, it can inhibit the hydroxylation of HIF-1 and give rise to activation of this transcription factor.

HIF-1 target genes are involved in erythropoiesis, angiogenesis, and energy metabolism. In a hypoxic environment, these genes have a lot of critical functions in controlling cellular homeostasis, which makes these genes involved in radio- and chemotherapy resistance of tumors, resulting in tumor survival and more aggressive phenotypes^14–16^. Several other genes, as mitotic kinase family (Aurora A, B, and C), which are associated with tumor enhancement and cancer progression, are also influenced by hypoxia. Therefore, these genes are new targets for treatment strategies^17–19^.

Although several different mechanisms have been found, one of the most dangerous problems in cancer patients is the drug resistance that frequently occurs and has a bad prognosis^20–22^. Some of these mechanisms are the prevention of the drug from entering in to the cell, pumping the drug out of the cell, enzymatic inactivation of drug, mutation or alteration of drug targets, apoptosis or senescence defectiveness, and deficient repair mechanisms. Drug resistance is the primary cause of unsuccessful treatment in patients with acute leukemia^23^. Multi Drug Resistance (MDR) is a well-known protein that is one of the primary pumps which can actively excrete drugs from the cell^24^. MDR-Associated Protein (MRP) acts like MDR toward excreting drugs out of the cell and when it is overexpressed in acute leukemia, tumoral cells show resistance against several classes of drugs such as anthracyclines, etoposide, and vinca alkaloids^25^. In patients for whom MRP was low, overall survival was better than patients with moderate or higher MRP expression^26^. Another protein that is reported to be involved in MDR phenotype is a newly characterized one and is called Breast Cancer Resistance Protein (BCRP, ABCG2) that is a member of ABC transporter family and seems to develop resistance against commonly used AML drugs^27^.

In order to have a better understanding of how the hypoxia and MSCs affect leukemia cells, the effects of the hypoxia and MSCs on MOLT-4 cells (ALL cell line) were studied. The proliferation rate, apoptosis, cytotoxicity and expression profiles of the genes involved in the drug resistance process that have very important functions in leukemic cells’ biological behavior were investigated.

## Materials and Methods

### Cell culture

The MOLT-4 cell line was purchased from Pasteur Institute Cell Bank (Tehran, Iran) and was cultured in RPMI 1640 (Gibco Laboratories, Grand Island, NY). Bone marrow MSCs (positive for CD44, CD90, STRO-1 and Negative for CD14, CD19, CD146) were purchased from Royesh Stem Cell Biotechnology Institute Cell Bank (Tehran, Iran) and were cultured in DMEM (Dulbecco’s Modified Eagle Medium) (Gibco Laboratories). Both media contained 10% fetal bovine serum (FBS) (Gibco Laboratories) and were incubated at 37°*C* humidified atmosphere in 5% CO_2_ incubator.

### Determining the dose of CoCl_2_ cytotoxicity

HIF1 induction *via* CoCl_2_ is dose-dependent. To reach the maximum levels of HIF1 without significant cell death, the CoCl_2_ induced cytotoxicity was assessed *via* cell counting by Trypan blue. To evaluate the CoCl_2_ cytotoxicity on MOLT-4 cells, different concentrations of cobalt (0, 25, 50, 100, 150, and 200 *μM* CoCl_2_) were employed.

### Co-culture of MOLT-4 cells with MSCs

Second passage MSCs were seeded in plates containing DMEM at a density of 5×10^4^ *cells/cm*^2^. The medium was changed every three days, till the MSCs feeder layer reached confluence. Subsequently, MOLT-4 cells in 2×10^6^ number were added into supernatant and incubated for 6 and 24 *hr*.

### Hypoxic treatment

2×10^6^ MOLT-4 cells, with or without MSCs, were treated with 100 *μM* CoCl_2_ in 5% CO_2_ incubator at 37°*C* for 6 and 24 *hr*.

### RNA extraction and cDNA synthesis

After removing the medium from plates, MSCs were adherent and MOLT-4 cells were suspended and in the medium just containing MOLT-4 cells, total RNA from leukemic cells was extracted using RNX-Plus solution kit (Sinaclon, Tehran, Iran). After cell lysis (first step based on manufacturer’s instruction), the products were stored at −70°*C* and thawed when RNA extraction was needed. High capacity kit (Bioneer, Alameda, CA) was used to produce single-stranded cDNA from the extracted RNA.

### Real-Time Quantitative Reverse Transcription Polymerase Chain Reaction (qRT-PCR)

The SYBR1 Green PCR Master Mix (Takara, Clontech, Japan) was used to determine the mRNA levels of BAX, BCL-2, MDR-1, and BCRP genes. The analysis of melting curves was performed using real-time PCR system (Rotor Gene 6000, Corbett Life Science). The supplemental table 1 shows the primers used for BAX, BCL-2, MDR-1, MRP, BCRP, β-actin and GAPDH genes. β-actin and GAPDH were used as an internal control, and duplicate analysis was performed for all samples. The list of the primers is given in table 1.

**Table 1.** Summary of primer sequences. All primer sequences are presented in 5′ to 3′ orientation

**Gene**	**Sequences (5′-3′)**	**Amplicon length (*bp*)**	**Annealing Temp (°*C*)**
**MDR1**	F: ATTGCTCACCGCCTGTCCACC R: TGCTGATGCGTGCCATGCTCC	90	57
**MRP**	F: CGTGTTGGTCTCTGTGTTCCTG R: AGAAAGATGCTCTCTGGGTTTG	181	57
**BCRP**	F: GGAATCTTGGCTGAGGGTTTGG R: GATGATTCTGACGCACACCTG	169	59
**Bax**	F: TGCCAGCAAACTGGTGCTCA R: GCACTCCCGCCACAAAGATG	194	59
**Bcl2**	F: CCTGTCGATGACTGAGTACC R: GAGACAGCCAGGAGAAATCA	128	55
**βactin**	F: AGAGCTACGAGCTGCCTGAC R: AGCACTGTGTTGGCGTACAG	184	57
**GAPDH**	F: GGCGTGAACCACGAGAAGTATAA R: CCCTCCACGATGCCAAAGT	119	59

### Apoptosis analysis using annexin VFITC/PI

Cells undergoing apoptosis were identified by annexin V and propidium iodide staining (BD Pharmingen, USA) according to the manufacturer’s instructions. Briefly, the cells were washed twice with cold PBS and then were resuspended in 1X binding buffer at a concentration of 1×10^6^ *cells/ml*. Then, 100 *μl* of the solution (1×10^5^ cells) was transferred to a 5 *ml* culture tube. 5 *μl* of annexin V-FITC and 5 *μl* of PI were also added. Then, the cells were vortexed gently and incubated for 15 *min* at RT (25°*C*) in the dark. Finally, 400 *μl* of 1×binding buffer was added to each tube and they were analyzed using FACSCalibur flow cytometer (Becton-Dickenson, Mountain View, CA, USA) and FlowJo software.

### Statistical analysis

Our results were statistically analyzed by The SPSS v.19. Data statement was as means±SD. One-Way ANOVA was used to assess the observed statistical differences. The GraphPad Prism v.6 was employed for regression analysis of correlation and the response linearity (GraphPad Software Inc). Statistically significant data were considered for p<0.05.

## Results

### Cell toxicity assay of CoCl_2_ treated cells

**Figure 1.**
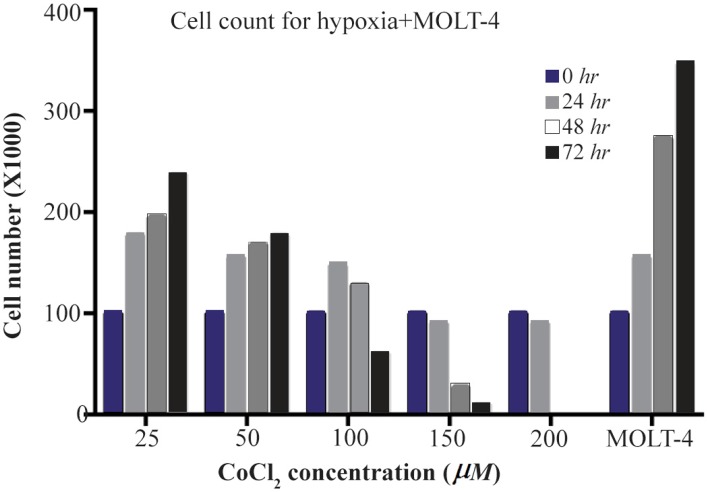
The MOLT-4 cells exposed to various doses of CoCl_2_ (0, 25, 50, 100, 150, 200 *μM*) during 0, 24, 48 and 72 *hr* time courses. In these periods, to detect the toxic dose of CoCl_2_, cells were counted using trypan blue in 1:1 ratio.

According to our results, with less than 100 *μM* doses of CoCl_2_, cell growth was observed at 48 and 72 *hr*, and at the dose of more than 100 *μM*, CoCl_2_ was fatal for cells in each of the designated times. So, the optimum dosage was fixed at 100 *μM* concentration of CoCl_2_ within 24 *hr* (Figure 1).

### Growth curve analysis of MOLT-4 cells co-cultured with MSC under the hypoxic condition

**Figure 2.**
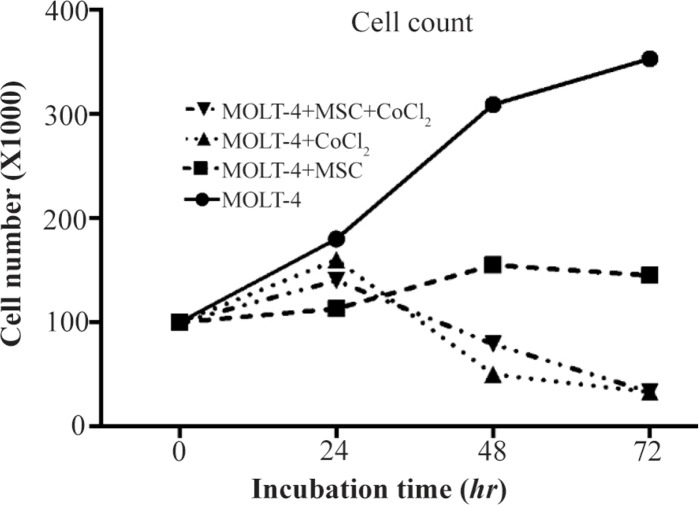
MOLT-4 cells cultured under different conditions (with MSC, with CoCl_2_, with MSC and CoCl_2_) counted by trypan blue at 0, 24, 48, 72 *hr*.

Cobalt exposed (100 *μM*, 24, 48, and 72 *hr*) and untreated MOLT-4 cells were cultured in mutual numbers on 6 *cm* cell culture plates. After 24, 48, and 72 *hr*, the cells of each plate were counted using trypan blue. The MOLT-4 cells were cultured under different conditions (with MSCs, with CoCl_2_, with MSCs and CoCl_2_) and were counted by trypan blue in 1:1 ratio at 24, 48, and 72 *hr*. Our findings revealed that MOLT-4 cells with MSC and CoCl_2_ alone, and MSC along with CoCl_2_ had inhibitory effects on growth of them in 24 *hr* (Figure 2).

### Comparison of BAX and BCL2 gene expression levels in MOLT-4 cells co-cultured with MSC under hypoxic condition

BAX and BCL2 were evaluated to determine apoptotic and anti-apoptotic changes in different conditions (MOLT-4+MSC, MOLT-4+CoCl_2_ and MOLT-4+MSC+CoCl_2_). BCL2 expression was increased in the presence of MOLT-4+MSC and MOLT-4+CoCl_2_ and was the highest in MOLT+MSCs+CoCl_2_ (p<0.05). However, BAX mRNA expression level did not differ significantly between different groups (Figures 3A and 3B).

**Figure 3A.**
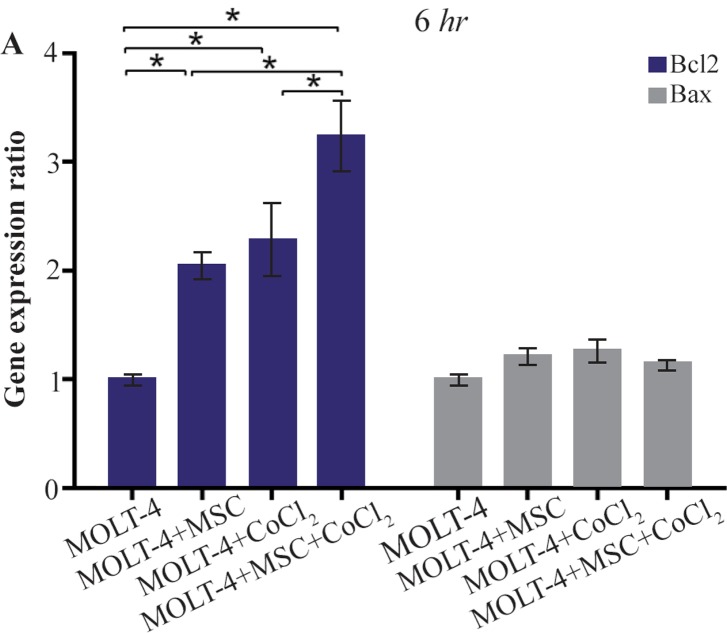
Real Time-PCR data for BAX and BCL2 expression in MOLT-4 cells under CoCl_2_ and hypoxia with and without MSC. (A) BAX and BCL2 expression levels were analyzed by Real Time-PCR in MOLT-4 cells under CoCl_2_ (100 *μM*) with MSC. RNA was extracted at 6 *hr* following 100 *μM* CoCl_2_ exposure. Data is presented as means±SD of three independent experiments. * Statistically significant difference compared to the respective data of control (untreated cells), p<0.05.

**Figure 3B.**
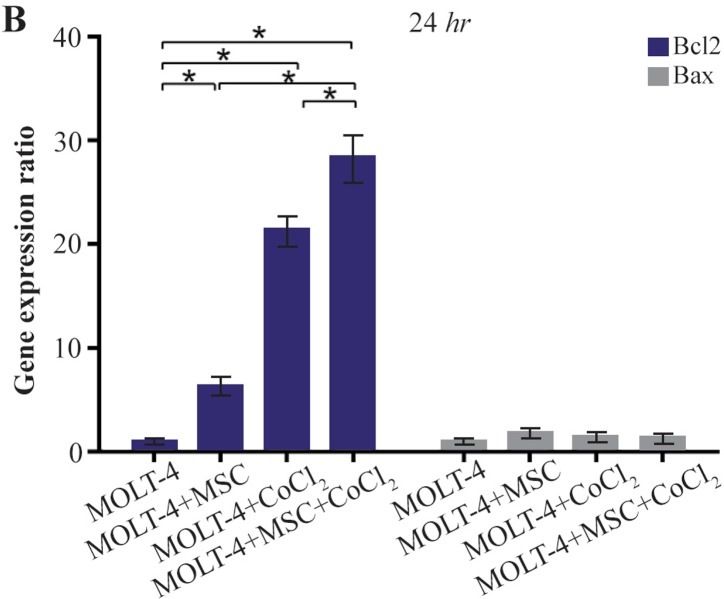
Real Time-PCR data for BAX and BCL2 expression in MOLT-4 cells under CoCl_2_ and hypoxia with and without MSC. (B) BAX and BCL2 expression levels were analyzed by Real Time-PCR in MOLT-4 cells co-cultured with MSC. RNA was extracted at 24 *hr* following 100 *μM* CoCl_2_ exposure. Data is presented as means±SD of three independent experiments. * Statistically significant difference compared to the respective data of control (untreated cells), p<0.05.

### Comparing the multiple drug resistance genes expression levels in MOLT-4 cells co-cultured with MSC under the hypoxic condition

MDR1, MRP, and BCRP were evaluated to determine the expression level changes of drug resistance genes in different conditions (MOLT-4+MSC, MOLT-4+CoCl_2_, and MOLT-4+MSC+CoCl_2_). MDR1 expression was increased in the presence of MOLT-4+MSC and MOLT-4+CoCl_2_ and was the highest in MOLT-4+MSC+CoCl_2_ (p<0.05).

BCRP expression was increased in the presence of MOLT-4+MSC and MOLT-4+CoCl_2_ and was the highest in MOLT-4+MSC+CoCl_2_ (p<0.05). However, MRP mRNA expression level did not differ significantly between different groups (Figures 4A and 4B).

**Figure 4A.**
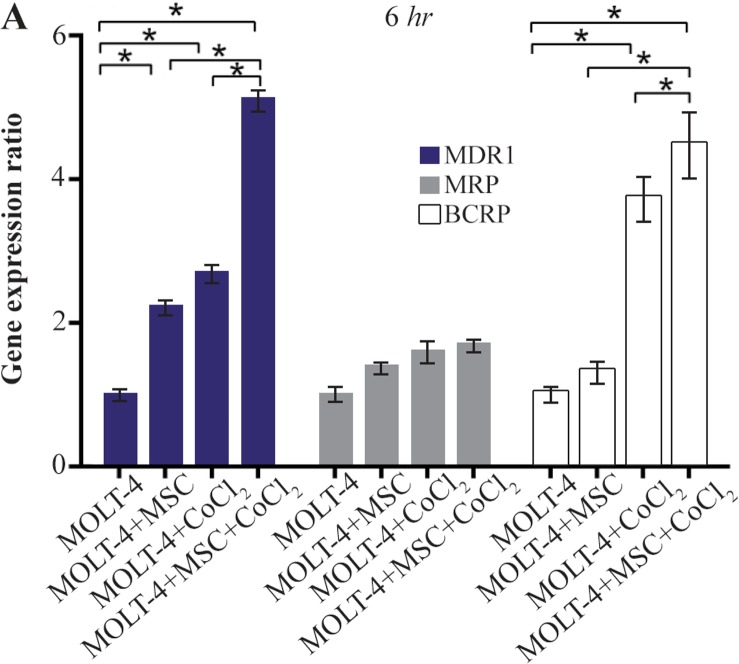
Real Time-PCR data for MOLT-4 cells MDR1, MRP, and BCRP genes expression under CoCl_2_ and hypoxia with and without MSC in different time courses. (A) MDR1, MRP and BCRP genes expression levels were analyzed by Real Time-PCR in MOLT-4 cells under CoCl_2_ (100 *μM*) with MSC. RNA was extracted at 6 *hr* following 100 *μM* CoCl_2_ exposure. Data is presented as means±SD of three independent experiments. * Statistically significant difference compared to the respective data of control (untreated cells), p<0.05.

**Figure 4B.**
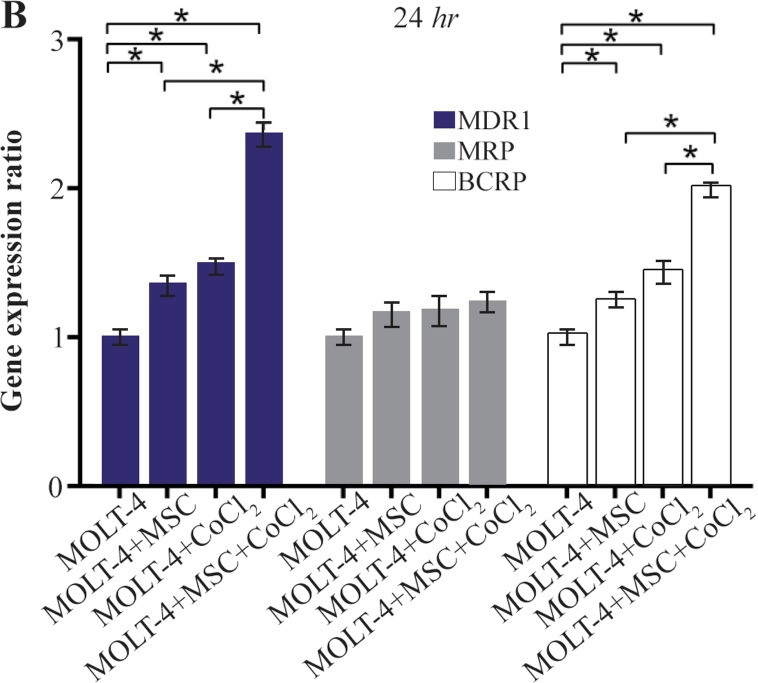
Real Time-PCR data for MOLT-4 cells MDR1, MRP, and BCRP genes expression under CoCl_2_ and hypoxia with and without MSC in different time courses. (B) MDR1, MRP and BCRP genes expression levels were analyzed by Real Time-PCR in MOLT-4 cells cocultured with MSC. RNA was extracted at 24 *hr* following 100 *μM* CoCl_2_ exposure. Data is presented as means±SD of three independent experiments. * Statistically significant difference compared to the respective data of control (untreated cells), p<0.05.

### Hypoxia-induced apoptosis of MOLT-4 cells co-cultured with MSC

Hypoxia-induced apoptosis of MOLT-4 cells co-cultured with MSC was assessed using annexin V/PI staining. Treatment of MOLT-4 cells co-cultured with MSC with 100 *μM* CoCl_2_ resulted in a statistically significant increase in the number of apoptotic cells after 24 *hr*. After 24 *hr* of CoCl_2_ treatment, apoptotic cells constituted about 20.80±0.66, 11.91±0.95, 8.10±0.44, and 2.21±0.42% of the totally measured cell population, that is MOLT-4, MOLT-4+MSC, MOLT-4+CoCl_2_ and MOLT-4+MSC+CoCl_2_, respectively (Figures 5A and 5B).

**Figure 5A.**
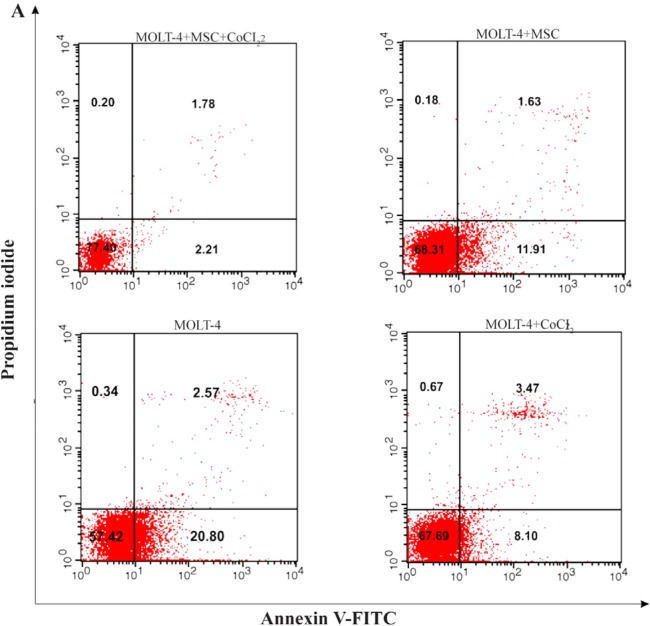
Recognition of apoptosis in MOLT-4 cells co-cultured with MSC treated with CoCl_2_. Figure 5A depicts a representable sample of assessment of apoptosis using Annexing-V staining and PI. (A) Change of phosphatidylserine externalization induced by CoCl_2_ in MOLT-4 cells cocultured with MSC (flow cytometry analysis, Annexin V-FITC/PI staining). MOLT-4 cells were incubated with 100 *μM* CoCl_2_ for 24 *hr*. The data presents the three independent experiments. Annexin V positive/PI negative cells (Annexin V+/PI−) showed in the bottom right quadrant of each dot plot signifies cells corresponding to early apoptosis, whereas the Annexin V positive/PI positive (Annexin V+/PI+) cells showed in the upper right quadrant signifies cells corresponding to late apoptotic/necrotic cells.

**Figure 5B.**
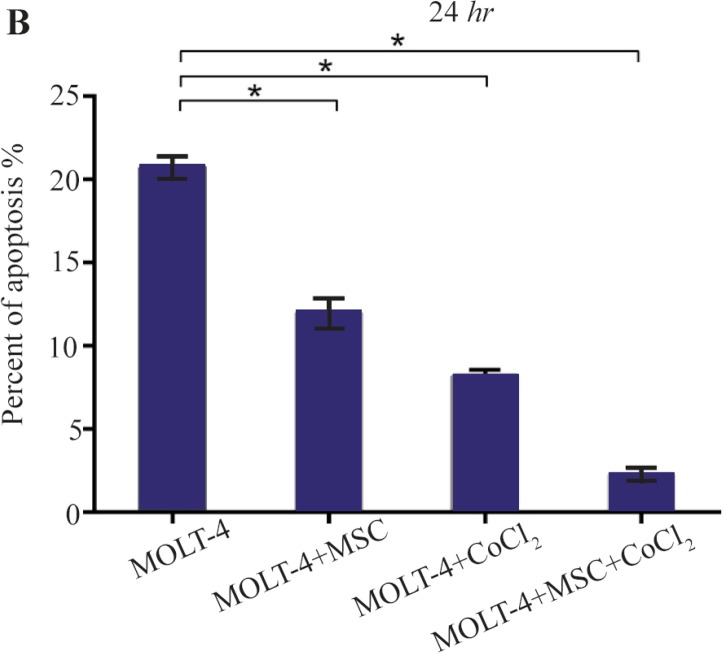
Recognition of apoptosis in MOLT-4 cells co-cultured with MSC treated with CoCl_2_. (B) Apoptotic cells Percentage (demonstrating phosphatidylserine externalization) induced by CoCl_2_ (flow cytometry analysis, Annexin V-FITC/PI staining). MOLT-4 cells co-cultured with MSC were incubated with 100 *μM* CoCl_2_ for 24 *hr*. Data is presented as means±SD of three independent experiments. * Statistically significant difference compared to the respective data of control (untreated cells), p<0.05.

## Discussion

Today, the hypoxia and drug resistance are considered to play roles in the progression and growth of tumor. There are clear shreds of evidence about the positive effect of HIF1 on the proliferation of leukemic cells, and it is one of the key regulators of the cell response to hypoxia^28,29^. It seems that hypoxia is a positive regulator of gene expression than a negative one and HIF1 might play a fundamental role in the regulation of the genes related to the hypoxia^30^. In the field of studies on ALL cells, it has been suggested that mesenchymal cells in co-culture with these cells led to some changes in their signaling process. Some studies have demonstrated that MSCs have inhibitory effects on proliferation of ALL cells in the co-culture experiments^1,31^. Others have reported that BCL2 gene which was significantly upregulated may play a role in the mechanism of the drug resistance induced by MSCs in the leukemic cells. Our results also showed that in co-culture of MOLT-4 cells, the BCL2 gene expression was increased, whereas BAX gene expression did not show significant changes. Our observations also confirmed previous studies and revealed the inhibitory effect of MSCs on MOLT-4 cell growth^1,32^. Besides, the impact of hypoxia was examined on MOLT-4 cells co-cultured with MSCs. Our results showed that the hypoxia induced by 100 *μM* CoCl_2_ significantly increased the expression of BCL2 gene, whereas the BAX gene expression displayed no significant alteration. Our outcomes presented that hypoxia, as well as co-culture with MSCs, leads to an increased expression of BCL2 gene in MOLT-4 cells. Unsurprisingly, the co-culture of this cancer cell line accompanied by hypoxia had the greatest increase in the BCL2 gene expression.

Several studies have been done on ALL lineage cells co-cultured with MSCs to explore the changes in the expression of drug resistance genes, which represents the influence of co-culture as mentioned above on the expression pattern of drug resistance genes^33–35^. In this study, an attempt was made to evaluate the changes in the gene expression pattern of the drug resistance genes including MDR1, MRP, and BCRP in MOLT-4 cells co-cultured with MSCs under the hypoxic condition. It is important that the MDR1 and MRP genes are targets for transcription factor HIF1^36^. Our findings revealed that MDR1 and BCRP genes expression levels significantly increased when MOLT-4 cells were co-cultured with MSC in the hypoxic condition, whereas the MRP gene expression did not change significantly in none of the studied situations. It has been demonstrated that the hypoxia increases the expression of anti-apoptotic protein BCL-2, which can result in the drug resistance of leukemic cells and treatment failure^37^. MSCs have a potential to retard the cell cycle, prevent the proliferation and reduce the apoptosis in leukemic cells. So, MSCs shield these cells against unfavorable conditions and eventually maintain their viability. The data obtained from flow cytometry studies revealed a decline in MOLT-4 cells apoptosis affected by MSCs and hypoxia. Considering the increase of the BCL2 gene expression during the MOLT-4 cells co-cultured with MSCs under the hypoxic condition, the apoptosis lowering was expectable.

A limitation of the present study was using CoCl_2_ as a hypoxia mimetic agent, that increases HIF1 expression and its DNA binding activity, instead of a low oxygen environment^38^. Obviously, it doesn’t induce true hypoxia due to the toxic influence of CoCl_2_ on the cells and it is expectable to get the conflicting results compared with the real hypoxic condition that can cause numerous various changes in the cell biology.

Of course, because of the different study designs, cell line class, oxygen tension, and hypoxia duration, there are disagreements in determining whether CoCl_2_ acts as a supportive or suppressive agent for leukemic cells development or not^38–42^.

## Conclusion

Our results showed that the hypoxia induced by CoCl_2_ has an inhibitory effect on proliferation of ALL lineage MOLT-4 cells co-cultured with MSCs that can be a result of CoCl_2_ toxicity rather than the effect of hypoxia. Also, hypoxia and MSCs can contribute to increased expression of drug resistance genes and less apoptosis in ALL cells. Hence, the induction of anti-apoptotic proteins expression impressed by hypoxia and MSCs can be involved in the leukemic cells’ drug resistance phenomenon and subsequently in the treatment failure. Further studies are required to demonstrate the role of hypoxia and MSCs on the biological behavior of ALL cells, which may lead to particular treatment outcomes.
